# The design of consumer behavior prediction and optimization model by integrating DQN and LSTM

**DOI:** 10.1371/journal.pone.0327548

**Published:** 2025-07-02

**Authors:** Na Liu, Dajiang Hu

**Affiliations:** School of Business, Chongqing City Management College, Chongqing, China; Sri Krishna College of Engineering and Technology, INDIA

## Abstract

Amidst the rapid evolution of e-commerce and the growing abundance of consumer shopping data, accurately identifying consumer interests and enabling targeted outreach has become a critical focus for merchants and researchers. This study introduces the RL-Trans framework, an innovative approach integrating Deep Reinforcement Learning (DQN) with Transformer to capture and analyze consumer interests intelligently. By leveraging consumer profiles and transactional records, the RL-Trans framework dynamically adapts to evolving consumer behavior, enabling personalized interest propagation. The framework initially employs a Transformer network to process consumer behavioral data using a multi-headed attention mechanism, It then integrates DQN to optimize the model culminating in an enhanced prediction layer that refines consumer interest analysis. Experimental results demonstrate the framework’s superior performance to conventional LSTM-based approaches, while achieving competitive efficacy relative to state-of-the-art methods. This study advances academic discourse by introducing a novel perspective and methodology for consumer behavior analysis. It provides theoretical foundations and practical insights for enhancing personalized services and marketing strategies in e-commerce.

## 1. Introduction

The domain of electronic commerce and its associated analysis of consumer behavior has witnessed swift evolution and numerous significant transformations since the proliferation of Internet technology. In its nascent stages, electronic commerce primarily focused on erecting electronic marketplaces and crafting online transaction systems. Simultaneously, consumer behavior analysis heavily leaned on rudimentary visit metrics and fundamental user satisfaction surveys [1]. However, with the march of technology and the dawn of the big data epoch, electronic commerce has embarked upon a novel phase characterized by personalized recommendations and tailored services. Meanwhile, the realm of consumer behavior analysis has progressively integrated more intricate data mining methodologies and machine learning algorithms, aiming to glean valuable insights into consumer preferences and behavioral trends from vast reservoirs of user data. Moreover, the ascent of social media and mobile Internet has introduced fresh user interaction points and founts of data to electronic commerce, propelling the advancement of social and mobile commerce. These shifts necessitate consumer behavior analysis to adeptly capture user interactions across platforms and dimensions to acclimate to the rapid fluctuations in user consumption patterns [2]. Furthermore, with the maturation of artificial intelligence technologies, particularly the adoption of deep learning and reinforcement learning, consumer behavior analysis has commenced its trajectory toward loftier objectives such as real-time dynamic analysis and prognostication, as well as the optimization of personalized recommendation algorithms, all in pursuit of refining user profiles with precision and enhancing the efficacy of recommendation systems.

Machine learning and deep learning assume pivotal roles in the scholarly realm of e-commerce and consumer behavior analysis, propelling the theoretical advancement of this domain and significantly enhancing the efficacy and efficiency of practical implementations [3]. By assimilating and distilling valuable insights from extensive troves of user interaction data, these methodologies empower enterprises to gain deeper insights into consumer needs and preferences, thereby facilitating the formulation of more bespoke marketing strategies and product recommendation systems. Machine learning techniques such as classification, regression, and cluster analysis prove instrumental in delineating consumer cohorts, prognosticating user conduct, and scrutinizing market dynamics [4]. Deep learning methodologies, notably Convolutional Neural Networks (CNNs), Recurrent Neural Networks (RNNs), Long Short-Term Memory Networks (LSTMs), and more recently, Transformer Networks, find widespread application in intricate tasks like discerning complex consumer behavior patterns, conducting sentiment analysis, predicting user interests, and tailoring recommendations, owing to their robust feature acquisition and sequence modeling prowess. By meticulously scrutinizing data streams encompassing users’ browsing histories, purchase logs, review contents, and social network engagements, these approaches adeptly discern users’ latent needs and shifts in their preferences, furnishing formidable data-driven support for e-commerce platforms to deliver enhanced consumer experiences. As the integration of reinforcement learning in sequential decision-making quandaries matures, its significance in consumer behavior analysis and e-commerce burgeons. By simulating decision-making paradigms in commercial contexts, reinforcement learning aids models in continually refining decision strategies, effectuating self-adjustment and optimizing dynamic recommender systems and intelligent marketing tactics, thereby further augmenting user satisfaction and economic yields for e-commerce enterprises [5].

E-commerce and consumer behavior analysis has entered a novel epoch that is underscored by data-centric, intelligent, personalized offerings. This juncture not only accentuates the analysis of user data through sophisticated algorithms but also emphasizes the development of intelligent frameworks capable of real-time market responsiveness and adaptable adjustment to evolving user requisites. Machine learning and deep learning technologies are significant for studying of e-commerce and consumer behavior analysis [6]. They not only enable enterprises to gain profound insights into the market and consumers at both macro and micro levels and wield considerable influence in personalized service provision, product recommendation, market prognostication, and user experience enhancement, among other facets [7]. Intelligent consumer behavior analysis facilitates enterprises in comprehending and fulfilling consumers’ exigencies more effectively, thus conferring competitive advantages amidst fervent market competition. Therefore, this paper delves into the backdrop of consumer behavior analysis and employs a deep learning time series network for user interest analysis. The specific contributions outlined in this paper are as follows:

(1) This paper confronts the challenge of analyzing consumer behavioral tendencies and interests, achieving intelligent prognostication of consumer interests by leveraging their browsing history data and corresponding interest categorization as the foundational dataset.
(2) The construction of the framework has been accomplished through the fusion of transformer architecture and reinforcement learning, enabling the recognition of consumer interests and the dissemination of pertinent content.
(3) Through rigorous testing on both public datasets and proprietary data, the performance of the proposed RL-Trans framework in this study markedly surpasses traditional analytical methods, demonstrating superior efficacy in intelligently driving user interest engagement.

The rest of the paper is arranged as follows: related works for consumer analysis based on machine learning and deep learning are introduced in Section 2; Section 3 establishes the RL-Trans model. Experiment results and related analysis are detailed described in Section; Section 5 in the Discussion. The conclusion is drawn at last.

## 2. Related works

### 2.1 Consumer behavior analysis based on machine learning methods

Sılahtaroğlu et al. [8] gathered data on consumers’ past consumption behaviors alongside statistical insights, employing neural networks and decision trees to prognosticate the likelihood of consumer purchases. Webb et al. [9] devised a model delineating customers’ purchasing behaviors, addressing four pivotal facets: data preparation, data labeling, concept transfer, and intricate calculations to forecast consumer purchasing patterns based on these aspects. ZUO et al. [10] scrutinized supermarket data, demonstrating superior results with the support vector machine model through comparative analyses. Chang et al. [11] established a model akin to traditional statistical methods to predict purchase probabilities. Guadagni et al. [12] assembled a cohort of one hundred purchasers of generic items, utilizing logistic regression to gauge brand loyalty, thereby predicting sales figures. Cui et al. [13] employed support vector machines in conjunction with multinomial logistic regression for predictive analysis, offering a framework that contrasts support vector machines with contemporary modeling techniques, delineating their strengths and limitations. Potharst [14] harnessed neural network algorithms to construct a predictive model for repeat purchasing behaviors, leveraging the algorithm’s adeptness in accommodating nonlinear feature variables. Baesens et al. [15] employed Bayesian neural network algorithms to mitigate data overfitting issues in constructing predictive models.

The research above underscores the burgeoning interest in consumer analysis within e-commerce, particularly through machine learning methodologies. However, as the volume and complexity of data continue to burgeon, traditional machine-learning approaches are rendered somewhat obsolete. Consequently, the evolution of deep learning technologies has ushered in more intelligent methods capable of dynamic environmental interaction, offering promising solutions to these evolving challenges.

### 2.2 Consumer behavior analysis using deep learning methods

With the ongoing advancement of deep learning technology, leveraging this technology for time series analysis of user data has emerged as a crucial avenue for predictive consumer behavior analysis. Inspired by gated circuits, researchers have introduced enhancements such as input, output, and forgetting gates in models based on gating mechanisms like LSTM [16] and GRU [17] to mitigate such problems as vanishing and exploding gradients. Hidasi et al. [18] integrated the GRU model into recommendation systems, proposing the GRU4Rec model to capture dependencies among users’ behavioral histories, thereby enhancing recommendation efficacy. However, the sequential computation inherent in RNN and their variants constrain model parallelism, leading to challenges in handling longer sequence data, where issues like gradient vanishing and explosion persist, especially for sequences exceeding a length of 100. Addressing this, the Transformer model introduced by Vaswani et al. [19] leverages attention mechanisms to overhaul the computational structure, enabling parallel processing and effective handling of extended time-series data. This innovation marked a significant improvement over traditional RNN structures, particularly in tasks like language translation and text classification, catalyzing further advancements in sequence modeling. Zhou et al. [20] identified the need for distinct user interest representations for various candidate items, proposing the DIN model equipped with an Attention mechanism to extract sequence features comprehensively. An Activation Unit (AU) was also introduced to tailor user embedding vectors, generating the user’s Attention Score for each item. Kang et al. [21] leveraged the Transformer encoder to capture dependencies among users’ historical behaviors, thereby extracting interest features to predict the next item of interest. This approach yielded superior results in experimental evaluations, showcasing the efficacy of leveraging Transformer-based architectures in consumer behavior prediction tasks.

## 3. Methodology

### 3.1 Transformer network

**Fig 1:**
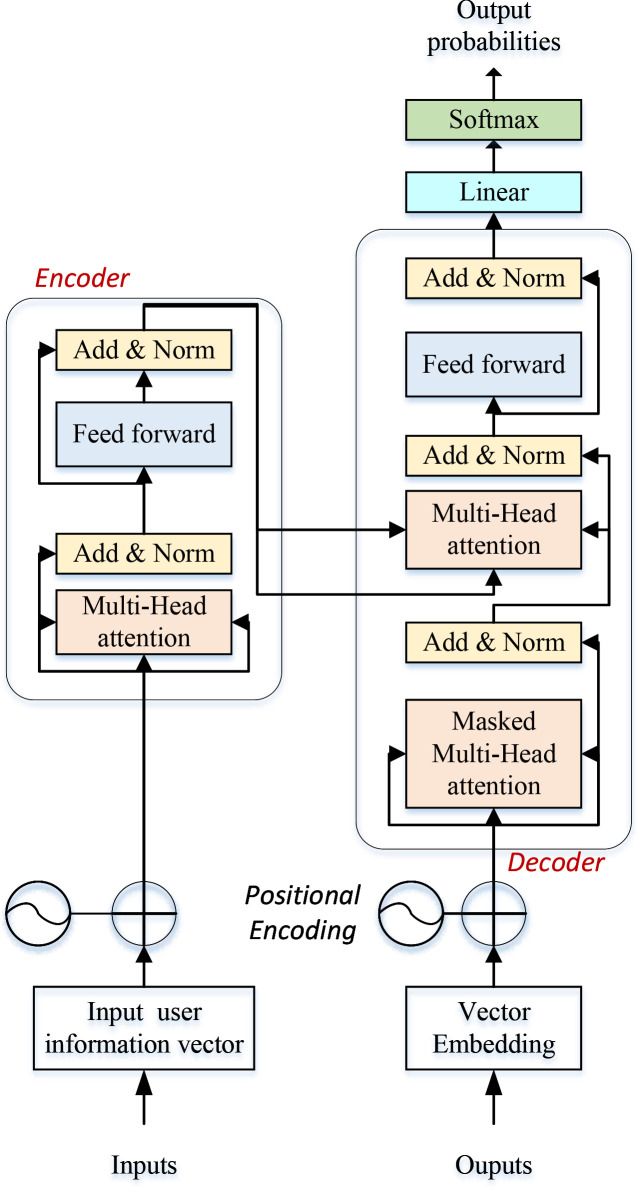
Framework for the transformer.

The Transformer model epitomizes a deep learning paradigm, harnessing the Self-Attention mechanism to tackle sequential data, thereby adeptly addressing long-range dependency challenges and facilitating efficient parallel data processing [22]. Preceding the advent of the Transformer, sequence-to-sequence (Seq2Seq) tasks like machine translation and text summarization heavily relied on RNNs and their derivatives, such as LSTM and GRUs. These models iteratively processed each sequence element to capture contextual information. However, RNNs encounter issues with vanishing or exploding gradients when handling lengthy sequences. Although LSTMs and GRUs somewhat alleviate this problem to some extent, the inherent sequential nature of RNNs impedes parallel processing, resulting in suboptimal training efficiency [23]. A schematic illustration of a typical Transformer network structure is presented in Fig 1:

In Fig 1, the TRANSFORMER network comprises four primary components: the self-attention mechanism, the multi-attention mechanism, positional encoding, and the encoding-decoding structure. Notably, the self-attention mechanism empowers the model to consider all elements within the sequence while processing each sequence element, ensuring that each element’s representation encapsulates information about the entire sequence. At the heart of the self-attention mechanism lies the computation of attention scores for each element concerning all other elements, followed by a weighted summation based on these scores to derive the new representation of each element, as depicted in Equation (1):


$$Attention(Q,K,V)=softmax\left(\frac{QK^{T}}{\sqrt{d_{k}}}\right)V$$ (1)

Where Q,K,V are the Query, Key, and Value matrices, respectively, and $d_{k}$ is the dimension of the key vector. This formula determines the weight of each value by calculating the similarity between the query and all the keys. The Transformer model amplifies the self-attention capability by utilizing a multi-head attention mechanism. Specifically, it conducts multiple parallel executions of the self-attention process, termed “heads,” wherein each head learns information about distinct sequence segments. Subsequently, the outputs of all heads are concatenated and processed through a linear layer. Given that the Transformer model inherently lacks the sequential processing capability of RNN, position encoding becomes imperative to furnish positional information to the model. Position encoding entails a vector that is added to the embedding vectors of each sequence element, thereby imbuing each element’s representation with positional information. The computational methodology for position encoding is delineated in equations (2) and (3):


$$PE_{\left(pos,2i\right)}=sin\left(pos/10000^{2i/d_{\text{model\textasciitilde{}}}}\right)$$ (2)

$${PE}_{\left(pos,2i+1\right)}=cos\left(pos/10000^{2i/d_{\text{model\textasciitilde{}}}}\right)$$ (3)

Where pos is the position and i is the dimension. The purpose of this is to allow the model to obtain the meaning of a word based on its relative or absolute position. The comprehensive process described above is embedded within the overarching structure of encoding and decoding, wherein the encoder handles input sequence processing while the decoder undertakes output sequence generation. In tasks like machine translation, the input sequence undergoes conversion into a sequence of contextual representations by the encoder, following which the decoder generates the output sequence based on these representations. Upon finalizing the model’s design through these constituent elements, the Transformer employs a normalization step to stabilize the training process at the output of each sub-layer (comprising the self-attention layer and the feed-forward neural network layer) in each module. The normalization formula is as follows:


$$LN(x)=\alpha ⊙\frac{x-\mu }{\sigma }+\beta$$ (4)

Where μ and σ are mean and standard deviation of α and β are the learnable parameters, and ⊙ denotes the element-by-element multiplication. In addition, the feedforward network in each encoding and decoding layer can be computed by Eq. (5):


$$FFN(x)=max\left(0,xW_{1}+b_{1}\right)W_{2}+b_{2}$$ (5)

Among them. $W_{1},W_{2},b_{1}$, and $b_{2}$ are learnable parameters. The feedforward network is applied independently, position by position, thereby enhancing the model’s nonlinear capabilities. Transformer incorporates residual connections between the inputs and outputs of each sublayer (self-attention and feedforward network), which are subsequently normalized using the following equation:


Output~=LN(Sublayer(x)+x) (6)

Residual connectivity aids in mitigating the issue of vanishing gradients in deep networks, thereby facilitating the training of deeper networks.

### 3.2 Reinforcement learning DQN

Reinforcement learning constitutes a pivotal branch of machine learning, focusing on how an intelligent agent learns an optimal behavioral strategy to maximize cumulative rewards through interactions with its environment. In this paradigm, the agent commences from an environmental state, executes actions, and receives rewards from the environment based on these actions, transitioning to a new state [24]. This iterative process continues, enabling the agent to gradually discern the optimal actions to maximize future rewards given the current state.

DQN, an acronym for Deep Q-Network, represents a reinforcement learning algorithm amalgamating deep learning and Q-learning. It primarily addresses the challenge of traditional reinforcement learning algorithms in coping with high-dimensional continuous state spaces. By leveraging a deep neural network as a function approximator, DQN effectively learns the action-value function, denoted as the Q function, by leveraging a deep neural network as a function approximator from high-dimensional perceptual inputs (e.g., pixels) [25]. In essence, DQN extends the Q-Learning framework by integrating deep learning techniques.

Within the Q-Learning framework, the fundamental update rule can be expressed as follows:


$$Q_{\text{new\textasciitilde{}}}(s,a)\leftarrow Q(s,a)+\alpha \left[r+\gamma \max_{a^{\prime }}\;Q\left(s^{\prime },a^{\prime }\right)-Q(s,a)\right]$$ (7)

Where Q(s,a) is in the state s The action to be taken in the state a, γ is the discount factor, which is used to regulate the importance of the reward at the end, and $\max_{a^{\prime }}\;Q\left(s^{\prime },a^{\prime }\right)$ is the next state $s^{\prime }$ the maximum of all possible actions in the next state Q value. In DQN, we use a deep neural network to approximate the Q-value function, and the network parameters are denoted by θ. The goal of DQN is to minimize the difference between the predicted Q-value and the target value. This goal can be achieved by minimizing the following loss function:


$$L(\theta )={𝔼}_{\left(s,a,r,s^{\prime }\right)\sim U(D)}\left[(y-Q(s,a;\theta ){)}^{2}\right]$$ (8)

Among them y is the target Q value, calculated as follows.


$$y=r+\gamma \max_{a^{\prime }}\;Q\left(s^{\prime },a^{\prime };\theta^{-}\right)$$ (9)

On this basis, experience playback and target network updates are carried out. The purpose of experience playback is to increase the diversity of training data and reduce the temporal correlation between samples, which is realized by transferring the transfer obtained from the interaction of the intelligent body with the environment $\left(s,a,r,s^{\prime }\right)$ is stored in the playback buffer D in the playback buffer, and when training, a batch of samples is randomly selected from D for learning. The target network is updated by periodically copying the parameters θ of the main network to the target network parameters θ*, which can be realized by soft updating, i.e., gradually mixing the weights of the main network into the target network.

### 3.3 RL-Trans for the user behavior analysis

**Fig 2:**
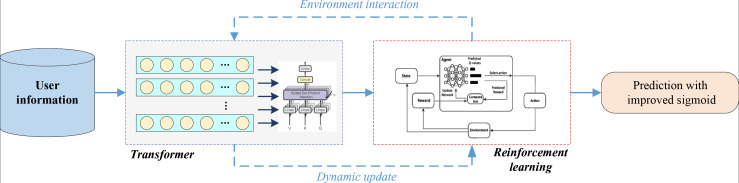
Framework for the RL-Trans.

After introducing both the Transformer network and the reinforcement learning, we constructed the RL-Trans network by integrating elements from both. The overall structure of the model is depicted in Fig 2:

The network initially processes various user information, preparing the corresponding vectors. Subsequently, leveraging the Transformer network, we execute combined feature extraction across diverse information through multi-head attention. Next, we employ the corresponding DQN network to facilitate dynamic interaction with the environment for reinforcement learning. Following these operations, we proceed with user interest recognition. Before computing the final result via sigmoid activation, we incorporate a DNN neural network structure to transform the concatenated feature information nonlinearly, enhancing the model’s nonlinear learning capabilities. The complexity of the model architecture can be adjusted according to specific scenarios by controlling the number of layers in the upper Transformer layer and the DNN layer. The DQN component in RL-Trans dynamically adapts to changes in consumer behavior by continuously updating its Q-values based on real-time interactions. To optimize this adaptation, a tailored reward function was designed to balance short-term engagement and long-term satisfaction. Anε-greedy exploration strategy was also implemented to ensure a trade-off between exploiting known consumer preferences and exploring new recommendation opportunities. The experience replay mechanism further enhances learning stability by storing past experiences and allowing the model to learn from diverse behavioral patterns, ensuring effective adaptation to evolving consumer interests.

In the training process outlined, we adopt the cross-entropy loss function. This loss function trains the model by minimizing the disparity between the model output and the actual label, thereby enhancing the model’s predictive classification capability. The cross-entropy loss function is calculated as shown in Equation (10):


$$L=-\frac{1}{N}\sum_{i=1}^{N}\;\sum_{c=1}^{C}\;y_{i,c}log\left({\overset{\circ }{y}}_{i,c}\right)$$ (10)

Where C is the total number of categories, and $y_{i,c}$ is an indicator function when the sample i belongs to the category c is 1 when the sample belongs to the category, and 0 otherwise, and ${\overset{\circ }{y}}_{i,c}$ is the model prediction that the sample i belongs to category c of the sample. The **enhanced prediction layer** in RL-Trans was designed to improve both accuracy and efficiency by incorporating **adaptive attention fusion** and **reinforcement-aware optimization** compared to conventional architectures. Instead of relying solely on standard dense layers, this layer integrates multi-head attention outputs with dynamically learned Q-values from the DQN module. This fusion ensures that both sequential consumer preferences and learned action policies contribute to the final prediction.

## 4. Experiment result and analysis

### 4.1 Dataset and experiment setup

The dataset selected for this paper is MovieLens-1M (MovieLens-1M: https://zenodo.org/records/5916501, https://doi.org/10.5281/zenodo.5916501) [26], a comprehensive collection of movie rating-related data obtained from the MovieLens movie recommendation system by GroupLens. It encompasses movie ratings, movie attributes, and user-related statistical features. Specifically, this paper utilizes one of the MovieLens-1M datasets comprising three files: ratings.dat, movies.dat, and users.dat. This dataset is a standard benchmark for testing and evaluating various recommender system algorithms, including collaborative filtering, content-based recommendation, and hybrid recommender systems. The MovieLens-1M dataset was preprocessed to ensure data quality and compatibility with RL-Trans. First, data was cleaned by removing duplicate entries and filtering users with fewer than 10 interactions to retain meaningful behavior patterns. Interaction sequences were then constructed by sorting user-movie interactions chronologically and segmenting sessions based on interaction gaps for reinforcement learning.

Feature engineering included one-hot encoding of user demographics, multi-hot encoding of movie genres, and learned embeddings for movie representations. Temporal features such as interaction recency were also incorporated. Finally, normalization techniques were applied, including scaling ratings to a [0,1] range and standardizing embeddings for stable model training. While proprietary data cannot be shared, we propose releasing a synthetic dataset that replicates key statistical properties, ensuring reproducibility and enabling further research in reinforcement learning-based recommendation systems. While a sizable dataset is suitable for addressing complex recommender system challenges, it is not as daunting as some larger datasets. The consumer behavior analysis in RL-Trans was conducted using a publicly available dataset, which underwent several preprocessing steps to ensure diversity and representativeness. First, data was cleaned to remove duplicates, inconsistencies, and missing values. Next, user interactions were tokenized and structured into sequential representations to capture behavioral patterns. Session-based segmentation was applied to enhance representativeness, preserving temporal dependencies in consumer actions. Additionally, categorical features such as product categories and user demographics were encoded for effective learning. Finally, the dataset was split using stratified sampling to maintain distribution consistency across training, validation, and test sets, ensuring that RL-Trans learns from a well-balanced and diverse set of consumer behaviors.

The primary objectives entail predicting users’ ratings for unseen movies based on their historical ratings and generating personalized movie recommendations based on users’ characteristics and movie attributes. For evaluation metrics, we regard each instance of user preference as a positive sample and assess the hit accuracy of predicting preference for specific products. This can essentially be viewed as a multi-source classification and recognition task, for which precision, recall, and F1 scores are selected as metrics to evaluate model performance. Additionally, we employ the hit rate (HR@k) to evaluate recommendation algorithms, which is calculated as shown in Equation (11):


$$HR@k=\frac{hitsnumber@k}{testdatannumber}$$ (11)

In addition to the more mainstream recommendation algorithms, our model also explores classic methods such as LSTM, GRU, and BI-LSTM, BI-GRU, alongside other established approaches. The analysis encompasses well-known algorithms including DSSM [27], SDM [28], DeepFM [29], and DIN [30]. Among them, the DSSM can be regarded as a basic benchmark model. DSSM (Deep Structured Semantic Model), used as a benchmark model, leverages deep neural networks to learn semantic representations of users and items, enabling recommendation through similarity-based vector matching. However, DSSM relies on fixed vector representations, making it less effective in capturing dynamic user interest changes and limiting its performance on long-sequence interactions. Additionally, it lacks a reinforcement learning mechanism, preventing adaptive optimization based on long-term user feedback. In contrast, RL-Trans, integrating Transformer and DQN, effectively captures long-range dependencies and dynamically optimizes recommendations, making it more suitable for complex consumer behavior modeling. Referring to the previous studies’ model establishement [31], the hyperparameters are set as follows Table 1:

**Table 1:** The hyperparameters setting.

Hyperparameter	Value
Learning Rate	0.0005
Optimizer	Adam
Transformer Depth	6
Number of Attention Heads	8
Hidden Layer Size	512
Dropout Rate	0.1
Batch Size	128
Training Epochs	50

### 4.2 The model comparison on the public dataset and ablation experiment

**Fig 3:**
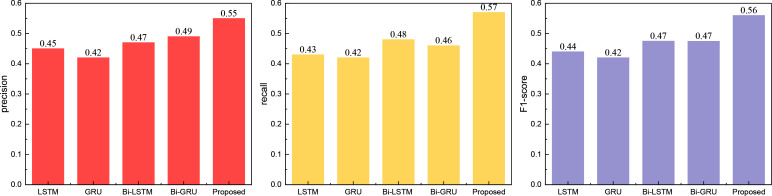
The comparison result for the classical method.

After establishing the respective models and defining the data indicators, we conducted a comprehensive analysis and comparison. Initially, we compared classical modeling methods by replacing the transformer module with LSTM, GRU, and other corresponding modules. The obtained results are presented in Fig 3:

In Fig 3, it is evident that the RL-Trans framework proposed outperforms traditional methods such as LSTM across all three categories of indicators: precision, recall, and F1-score. The improvement is notably significant, with all three indicators exceeding 0.5, representing a substantial enhancement over other methods.

**Fig 4:**
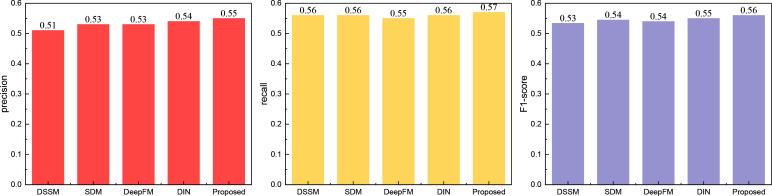
The comparison result for the currently used method.

Following the comparison of classical models, we evaluated the widely used push recognition recall model. The results are depicted in Fig 4:

Through Fig 4, it is evident that the RL-Trans method proposed maintains a distinct advantage over the more recent recognition recommendation algorithms. Across precision and recall metrics, all five methods achieve values exceeding 0.5, yet the performance of the RL-Trans method stands out as superior. This observation underscores the efficacy of the proposed method.

**Fig 5:**
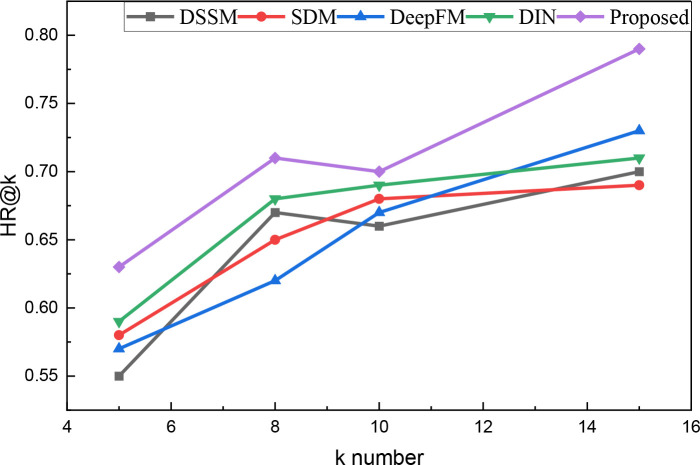
The HR@k result among different k.

Following comparing and optimizing of the recognition aspect, we proceeded to simulate the number of hotspot pushes. Subsequently, we analyzed the hit results under varying numbers of pushes, with the findings presented in Table 2 and Fig 5:

**Table 2:** The comparison result for the HR@k with different k numbers.

K number	DSSM	SDM	DeepFM	DIN	Proposed
5	0.55	0.58	0.57	0.59	0.63
8	0.67	0.65	0.62	0.68	0.71
10	0.66	0.68	0.67	0.69	0.7
15	0.7	0.69	0.73	0.71	0.79

As depicted in Fig 5, the hit rate of the method proposed consistently outperforms other push methods across different numbers of pushes. Notably, its overall results remain stable even when pushing 10 corresponding movies. This observation suggests that the proposed method delivers higher precision with lower standard requirements, thus effectively meeting the need for precise pushes.

### 4.3 The practical test for the RL-Trans and ablation experiment

**Fig 6:**
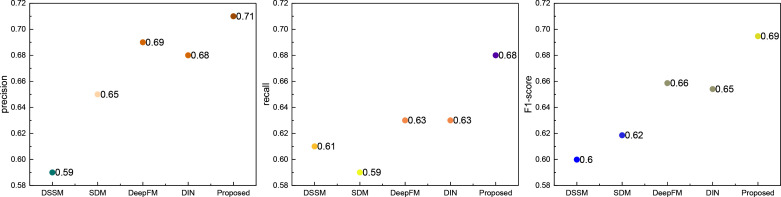
The method comparison on the self-established dataset.

Upon completing the model’s training of the model using the public dataset, we assessed its performance on a self-constructed dataset. The self-constructed dataset comprises shopping preferences and relevant shopping data of nearly 100 types of products from a total of 500 users on shopping websites in this region. Following meticulous data cleaning and feature extraction, we conducted further model analysis. The results obtained for precision, recall, and F1-score are depicted in Fig 6:

In Fig 6, it’s evident that the proposed method demonstrates superior recognition performance across all three evaluation metrics. When comparing precision indices, the recognition results of our method and the DeepFM method are relatively close, with values of 0.91 and 0.69, respectively.

However, in terms of recall indices, our method outperforms DeepFM significantly, indicating better stability and generalization performance.

**Fig 7:**
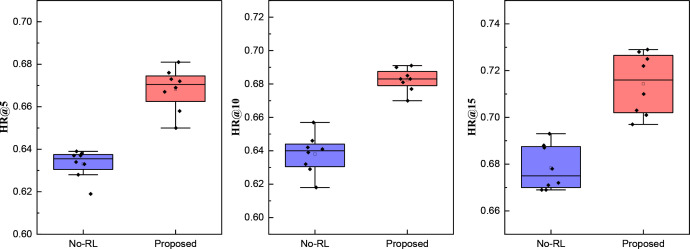
The ablation experiment result for the HR@k.

To further evaluate the model, we compared different push targets with and without reinforcement learning across various test subjects. The results are illustrated in Fig 7:

In Fig 7, we present the push hit results of RL-Trans under three different push quantity settings: 5, 10, and 15. The results indicate a clear pattern as the number of pushes increases, showing how different modules contribute to model performance.

Firstly, the Transformer module is crucial in capturing long-range dependencies, effectively modeling users’ long-term interest shifts and ensuring more globally consistent recommendations. Compared to traditional sequential models like RNNs or LSTMs, Transformer leverages the multi-head self-attention mechanism to focus on different time-step dependencies, making the push content more aligned with users’ potential preferences. Even without reinforcement learning, the Transformer maintains relatively stable recommendation accuracy, leading to a more evenly distributed performance across different push settings. However, Transformer alone lacks adaptability in dynamic environments, as it does not adjust its recommendation strategy based on long-term user feedback. We incorporate the DQN reinforcement learning module to address this limitation, which optimizes push strategies through interactive learning. DQN enables the model to adjust recommendations dynamically under different push quantities and user groups, learning the optimal sequence of recommendations for long-term rewards. Experimental results demonstrate that, across different push tasks (5, 10, or 15 pushes), RL-Trans with DQN consistently outperforms the Transformer-only variant, confirming that reinforcement learning enhances recommendation effectiveness and improves adaptability and generalization.

In summary, the Transformer module constructs robust feature representations and global interest modeling, while DQN further strengthens the model’s decision-making ability. This synergy enables RL-Trans to maintain superior performance across various push settings, delivering more precise consumer behavior predictions and personalized recommendations.

## 5. Discussion

For the consumer interest prediction problem, the RL-Trans framework proposed merges the strengths of Deep Reinforcement Learning (DQN) and the Transformer network, introducing an innovative approach to consumer interest push. Leveraging reinforcement learning’s prowess in sequence decision problems and the Transformer’s efficacy in processing lengthy sequence data, the RL-Trans framework achieves heightened recognition accuracy and push efficiency in consumer interest push endeavors. Unlike traditional time series processing methods such as LSTM and GRU, the RL-Trans framework adeptly captures and learns consumer behavioral patterns and preference changes more efficiently. Integrating Deep Q-Network (DQN) with the Transformer architecture in RL-Trans addresses the limitations of traditional LSTM-based approaches in consumer behavior analysis. LSTMs struggle with long-range dependencies and sequential bottlenecks, whereas Transformers capture global interactions more effectively. However, standard Transformers lack decision-making capabilities in dynamic environments. By incorporating DQN, RL-Trans enhances its ability to learn optimal action policies, enabling adaptive and personalized recommendations based on evolving user behaviors. This combination improves long-term dependency modeling and decision efficiency, overcoming the limitations of LSTM-based reinforcement learning methods. The Transformer’s Self-Attention mechanism enables it to grasp the dependency between any two points in a sequence, regardless of distance. This capability empowers the RL-Trans framework to comprehend and accurately predict consumers’ points of interest more accurately, facilitating more personalized and precise content push. The multi-head attention mechanism in the Transformer allows RL-Trans to capture diverse behavioral patterns by attending to different aspects of consumer interactions simultaneously. Unlike traditional sequential models, which process information step by step, multi-head attention enables parallel processing of multiple behavioral dependencies, identifying short-term preferences and long-term trends. This enhances the framework’s ability to analyze complex consumer behaviors, improving the accuracy of decision-making and personalized recommendations by considering various interaction contexts in a more granular and comprehensive manner. The automatic optimization of recommendation strategies through reinforcement learning techniques adjusts recommended content in real-time to accommodate shifts in user interests. It monitors user behavior over time to discern potential interest drift. This dynamic adjustment mechanism enables the RL-Trans framework to continually furnish recommendations aligned with users’ interests and requirements, rendering it more flexible and effective than conventional methods.

Furthermore, the RL-Trans framework demonstrates unique advantages compared to existing approaches such as DSSM and DIN, which have successfully understood user interests and enhanced recommendation accuracy. However, these methods primarily rely on static user behavior data and afford less consideration to user interests’ dynamic changes and long-term evolution. By amalgamating the policy optimization of reinforcement learning with the long-range dependency capture capability of the Transformer, the RL-Trans framework comprehensively grasps users’ immediate and long-term interests, thereby offering more dynamic and personalized push recommendations.

Integrating reinforcement learning and time series analysis for intelligent prediction and analysis of consumer behavioral habits can provide a deeper and more accurate understanding of consumer needs, enhancing the quality and efficiency of personalized recommendations. Through the combination of Reinforcement Learning and Transformer, the RL-Trans framework adeptly captures consumer behavioral patterns and interest preferences, elevating recommendation systems’ accuracy and user satisfaction. By continually tracking and analyzing consumer behavior over time, the RL-Trans framework enables companies to gain insights into the evolution of consumer demand and facilitates data-driven long-term user relationship management. Applying the RL-Trans framework advances personalized recommendation technology, offering e-commerce platforms innovative opportunities such as customized marketing and dynamic price adjustments. However, it’s crucial to rigorously adhere to data protection regulations to safeguard users’ privacy rights. Transparent data processing procedures and obtaining adequate user authorization are imperative. Moreover, given the dynamic nature of consumer behavior and the market environment, models should be periodically retrained and optimized to stay abreast of evolving consumer behavior and market trends. Additionally, while enhancing recommendation accuracy, it’s essential to maintain the naturalness and comfort of the user experience and mitigate user fatigue caused by excessive recommendations.

## 6. Conclusion

This paper introduces an innovative RL-Trans framework that integrates Deep Reinforcement Learning (DQN) and Transformer Networks to offer an efficient technical solution for consumer interest analysis and personalized push. Our framework dynamically learns and adapts to changes in consumer behavior, enabling personalized interest push by effectively leveraging consumers’ user information and shopping data. Leveraging the Transformer network, our RL-Trans framework employs the multi-head attention mechanism to process consumer behavioral data, followed by integration with DQN to optimize the model’s dynamic performance. This integration accurately completes consumer interest analysis through an improved prediction layer. In numerous experimental comparisons, our RL-Trans framework significantly outperforms traditional time series processing methods, such as LSTM in terms of accuracy and efficiency while demonstrating comparable or superior performance to current popular models. Moreover, the successful application of the RL-Trans framework underscores the substantial potential of deep learning and reinforcement learning in understanding and predicting complex consumer behavior patterns.

Future research endeavors will explore the generalization capability of the RL-Trans framework and extend its application across various e-commerce scenarios. We will also further refine the model structure and algorithms to enhance predictive performance and real-time response capabilities. Additionally, efforts will be directed toward establishing a more robust data support foundation for consumer interest analysis and personalized recommendations in e-commerce through data normalization and standardization initiatives.
